# TAVR With Patient-Specific Coronary Alignment for a Patient With an Anomalous Left Coronary Artery

**DOI:** 10.1016/j.jaccas.2026.108751

**Published:** 2026-06-03

**Authors:** Aekapat Phoksiri, Yusuke Kobari, Tau Hartikainen, Juhana Toimela, Gintautas Bieliauskas, Arif Azhar Khokhar, Ole De Backer

**Affiliations:** The Heart Center, Rigshospitalet, Copenhagen University Hospital, Copenhagen, Denmark

**Keywords:** anomalous coronary origin, coronary access, coronary alignment, implantation technique, TAVR

## Abstract

**Background:**

An anomalous coronary artery is rare in patients undergoing transcatheter aortic valve replacement (TAVR). Achieving coronary alignment when performing TAVR can be challenging, but it is essential to preserve future coronary access and redo-TAVR options, particularly in younger patients with a longer life expectancy.

**Case Summary:**

A 62-year-old patient with severe, symptomatic bicuspid aortic stenosis and an anomalous left coronary artery underwent TAVR without complications. TAVR with coronary alignment was achieved by relying on patient-specific fluoroscopic projections and meticulous preprocedural cardiac computed tomography (CT) planning.

**Discussion:**

An anomalous coronary anatomy can complicate TAVR planning and execution. Meticulous preprocedural planning based on cardiac CT is key in order to be successful and obtain patient-specific coronary alignment.

**Take-Home Messages:**

When treating younger patients with TAVR, identifying an anomalous coronary artery with an aberrant origin is important. TAVR with patient-specific coronary alignment in case of an anomalous coronary artery is feasible and requires meticulous preprocedural CT planning.

## Visual Summary

**Figure:**
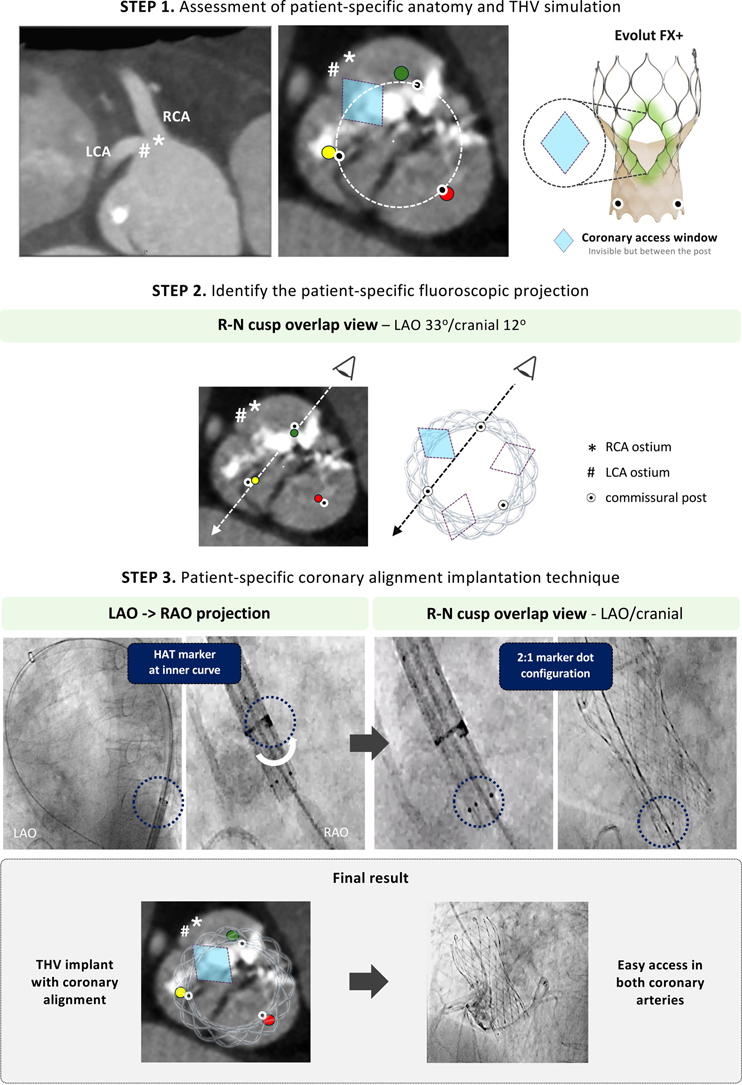

## History and Presentation

A 62-year-old man was referred for progressive exertional dyspnea categorized as NYHA functional class III. Physical examination revealed a systolic ejection murmur at the left upper parasternal border, consistent with severe symptomatic aortic stenosis.

## Past Medical History

The patient had multiple comorbidities, including arterial hypertension, dyslipidemia, prior disabling stroke with residual dense left hemiparesis and functional impairment, and severe chronic obstructive pulmonary disease (stage 3).

## Investigations

**Figure 1:**
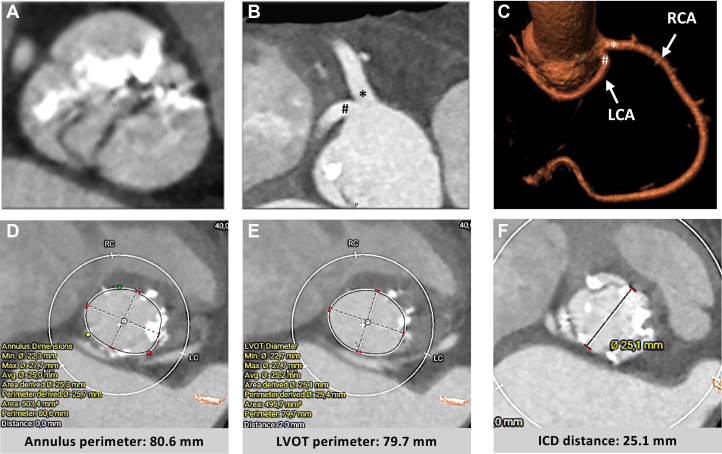
Preprocedural Computed Tomography Analysis (A) Calcified bicuspid aortic valve Sievers type 1 with raphe between the RCC and NCC. (B) Aberrant origin of the left coronary artery (#) originating from the RCC. (C) Three-dimensional CT reconstruction demonstrating a retroaortic course of the left coronary artery. (D) Aortic annulus dimensions. (E) Left ventricular outflow tract dimensions. (F) The intercommissural distance as measured 4 mm above the annulus. CT = computed tomography; ICD = intercommissural distance; LCA = left coronary artery; LVOT = left ventricular outflow tract; NCC = noncoronary cusp; RCA = right coronary artery; RCC = right coronary cusp.

Transthoracic echocardiography showed a left ventricular ejection fraction of 45% and severe aortic stenosis with a peak/mean transvalvular gradient of 90/55 mm Hg. Preprocedural computed tomography (CT) revealed a Sievers type 1 bicuspid aortic valve with right coronary–noncoronary (R-N) cusp fusion. The aortic annular perimeter was 80.6 mm, the left ventricular outflow tract perimeter was 79.7 mm, and the intercommissural distance measured 25.1 mm at 4 mm above the annulus and 25.7 mm at 8 mm above the annulus. An aberrant origin of the left coronary artery from the right coronary cusp was identified, with an anomalous retroaortic course (Figure 1, Video 1). There was no significant obstructive coronary artery disease.

## Management

The patient was discussed at the local heart team meeting. The younger age, presence of bicuspid aortic valve stenosis, and anomalous coronary artery favored surgical aortic valve replacement. However, the significant lung disease, together with the previous disabling stroke and frailty, were concerning for postoperative recovery and outcome. Therefore, after consultation with the patient, transcatheter aortic valve replacement (TAVR) was deemed the optimal treatment strategy.

Another key anatomical concern was the anomalous left coronary artery, with its ostium arising from the right coronary cusp and its course “wrapping” around the aortic annulus (Figure 1). With both coronary ostia close to the raphe and a relatively spacious aortic root, the risk of coronary obstruction was estimated to be low. Given these considerations a 29-mm Evolut FX+ (Medtronic) was selected, with pre- and postdilatation planned to facilitate and optimize transcatheter heart valve expansion. The hat marker of the delivery system, together with the 3 commissural dot markers found on the valve itself, would facilitate patient-specific coronary alignment, and finally, the large diamond-shaped cell of the latest generation Evolut FX+ platform would facilitate coronary access—a key consideration for this younger patient.

**Figure 2:**
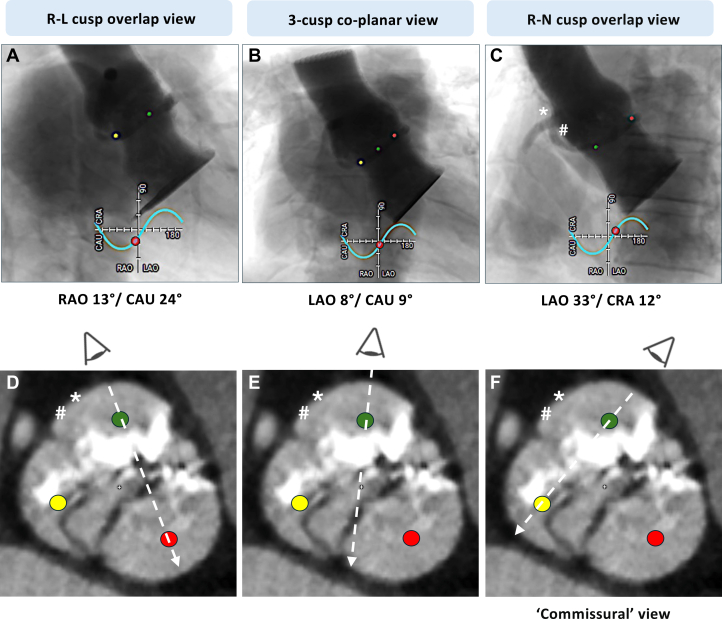
Computed Tomography–Derived Fluoroscopic Views (A to C) Three-dimensional CT reconstructed views. (D to F) Two-dimensional CT axial planes a few millimeters above the aortic annulus level. (A) and (D) show the R-L cusp overlap view. (B) and (E) show the 3-cusp coplanar view. (C) and (F) show the R-N cusp overlap view, also called the “commissural view” in case of a type 1 bicuspid aortic valve with R-N fusion. The yellow dot denotes the noncoronary cusp, the green dot denotes the right coronary cusp, and the red dot denotes the left coronary cusp. ∗ostium of the right coronary artery; #ostium of the left coronary artery. CAU = caudal; CRA = cranial; CT = computed tomography; LAO = left anterior oblique; RAO = right anterior oblique; R-L = right coronary–left coronary; R-N = right coronary–noncoronary.

**Figure 3:**
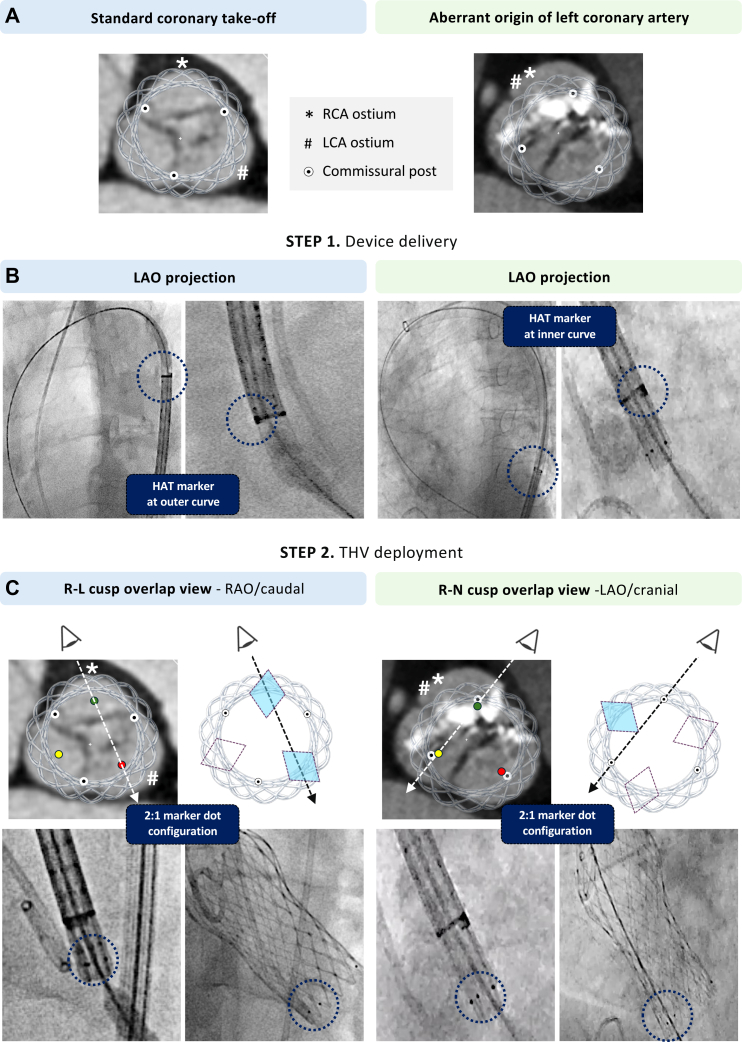
Coronary Alignment Implantation Steps With the Evolut FX+ System The left side shows how to obtain coronary alignment in case of a standard anatomy. The right side shows how to obtain coronary alignment in our patient with an anomalous left coronary artery. (A) Simulation of the desired Evolut FX+ orientation. (B) Position of the hat marker when crossing the aortic arch with the delivery system. (C) Desired Evolut FX+ orientation and marked dot configuration in the respective implantation views. ∗ostium of the right coronary artery; #ostium of the left coronary artery. LAO = left anterior oblique; LCA = left coronary artery; RAO = right anterior oblique; RCA = right coronary artery; THV = transcatheter heart valve.

On the other hand, given the patient's young age, it was considered a priority to strive for coronary alignment in order to facilitate future coronary access and redo TAVR. The standard CT-derived fluoroscopic views were determined to guide valve deployment (Figure 2). These projections are routinely used to assess implantation depth, while the right-left (R-L) cusp overlap view is also used to evaluate commissural alignment when implanting the Evolut prosthesis. In this particular patient with both coronary arteries taking off close to the R-N commissure/raphe, it could be simulated and anticipated that the R-N cusp overlap view would be useful for both achieving coronary alignment and visualizing the coronary takeoff on fluoroscopy (Figure 3).

In standard practice, the Evolut hat marker is oriented toward the outer curve of the descending and ascending aorta in a left anterior oblique projection when crossing the aortic arch in order to obtain commissural alignment. In this particular patient, we actually strived for the opposite: TAVR with patient-specific coronary alignment rather than generic commissural alignment (Figure 3A).

Using “reverse thinking,” we reasoned that—in this particular patient—the hat marker should be positioned toward the inner curve when crossing the aortic arch with the Evolut system in a left anterior oblique projection (Figure 3B, Video 2). Next, based on the CT planning, it could also be anticipated that a 2:1 marker-dot configuration should be obtained in the R-N cusp overlap view in order to obtain the desired Evolut FX+ orientation (Figure 3C, Videos 3 and 4), namely, an Evolut FX+ implantation with one of the large coronary access windows in front of both coronary ostia. This was executed exactly as planned, resulting in successful implantation of the Evolut FX+ prosthesis with virtually no invasive transvalvular gradient and no visible leak into the left ventricle with control aortography. Additionally, patient-specific coronary alignment was achieved as demonstrated by selective cannulation of both coronary arteries using a 6-F Judkins Right diagnostic catheter through the open diamond-shaped cell of the Evolut FX+ (Video 5).

## Follow-Up

After the procedure, transthoracic echocardiography showed a well-functioning transcatheter aortic valve prosthesis, with an aortic valve area of 2.1 cm^2^ and no evidence of paravalvular leak. A follow-up CT at 3 months after the procedure was planned. At the 30-day follow-up, the patient reported significant symptomatic improvement.

## Discussion

Anomalous coronary arteries are fairly uncommon (1%-7%)^1^ and rare in patients undergoing TAVR. Among approximately 40 reported TAVR cases with coronary anomalies, the most frequent finding is a left circumflex artery arising from the right coronary cusp (58%), followed by an aberrant origin of the left main from the right coronary cusp (21%), with coronary obstruction reported in 12.5% of cases.^2^ Preprocedural CT planning is critical to identify anatomical features associated with an increased risk of coronary obstruction. In addition to this risk assessment, striving for coronary alignment should be considered a key procedural step, particularly in younger patients, as it facilitates future coronary access and redo TAVR. Comprehensive knowledge of the underlying aortic and coronary anatomy, fluoroscopic implantation views and platform-specific features, and implantation techniques is essential to achieve successful patient-specific coronary alignment during TAVR,^3^^,^^4^ this even in the presence of a more complex underlying bicuspid aortic valve morphology.

## Conclusions

An anomalous coronary anatomy can complicate TAVR planning and execution. Meticulous preprocedural planning based on cardiac CT analysis, simulation, and “reverse thinking” is key in order to be successful and obtain patient-specific coronary alignment.

**Visual Summary:**
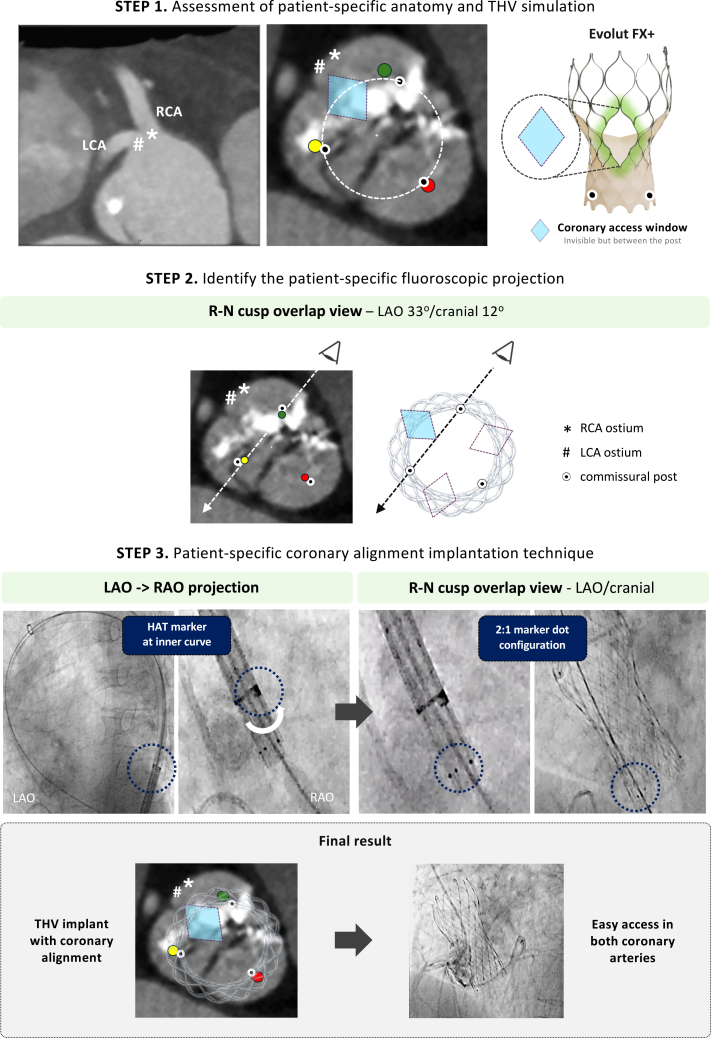
TAVR With Patient-Specific Coronary Alignment in Bicuspid Case With Anomalous Coronary Anatomy LAO = left anterior oblique; LCA = left coronary artery; RAO = right anterior oblique; RCA = right coronary artery; THV = transcatheter heart valve.

## Funding Support and Author Disclosures

The authors have reported that they have no relationships relevant to the contents of this paper to disclose.

**Take-Home Messages**

• When treating younger patients with TAVR, identifying an anomalous coronary artery with an aberrant origin is important.
• TAVR with patient-specific coronary alignment in case of an anomalous coronary artery is feasible and requires meticulous preprocedural CT planning.

## Appendix

Video 1 Preprocedural CT of the Bicuspid Aortic Valve Fly-through CT analysis demonstrating bicuspid aortic valve phenotype with right coronary cusp–noncoronary cusp fusion. Anomalous origin of the left coronary ostium seen originating from the right coronary cusp adjacent to the right coronary artery.

Video 2 Evolut Orientation for Patient-Specific Coronary Alignment The hat marker was positioned on the inner curve of the descending aorta and was tracked along the inner curve of the aortic arch (in an LAO projection).

Video 3 Implantation of Evolut FX+ After predilatation, a 29-mm Evolut FX+ prosthesis was implanted. Initial deployment shown in the intercommissural view (LAO/CRA). Implantation depth was assessed using 3-cusp coplanar view (LAO/CAU), followed by final release. After TAV deployment, 2 dots were positioned on left side of the screen (red arrow) in the intercommissural view (LAO/CRA).

Video 4 Final Angiographic Result of 29-mm Evolut FX+ Valve The valve was fully expanded, with low transprosthetic gradients and no paravalvular leak. (LAO/CRA projection shown corresponding to the intercommissural view).

Video 5 Coronary Cannulation After TAVR Cannulation of the common coronary ostia was achieved using a 6-F Judkins Right diagnostic catheter from the femoral access route. Cannulation was successfully achieved through the large diamond-shaped cell of the Evolut valve, which was positioned directly in front of the coronary ostium. The anomalous retroaortic left coronary artery and the right coronary artery are both visible.
